# The use of dried cerebrospinal fluid filter paper spots as a substrate for PCR diagnosis of the aetiology of bacterial meningitis in the Lao PDR

**DOI:** 10.1111/1469-0691.12260

**Published:** 2013-06-06

**Authors:** I Elliott, S Dittrich, D Paris, A Sengduanphachanh, P Phoumin, P N Newton

**Affiliations:** 1 Lao-Oxford-Mahosot Hospital-Wellcome Trust Research Unit (LOMWRU), Microbiology Laboratory, Mahosot HospitalVientiane, Lao PDR; 2 Centre for Tropical Medicine, Nuffield Department of Medicine, Churchill Hospital, University of OxfordOxford, UK; 3 Mahidol-Oxford Tropical Medicine Research Programme, Faculty of Tropical Medicine, Mahidol UniversityBangkok, Thailand

**Keywords:** Bacterial meningitis, cerebrospinal fluid, filter paper, Lao PDR, *Streptococcus pneumoniae*

## Abstract

We investigated whether dried cerebrospinal fluid (CSF) conserved on filter paper can be used as a substrate for accurate PCR diagnosis of important causes of bacterial meningitis in the Lao PDR. Using mock CSF, we investigated and optimized filter paper varieties, paper punch sizes, elution volumes and quantities of DNA template to achieve sensitive and reliable detection of bacterial DNA from filter paper specimens. FTA Elute Micro Card™ (Whatman, Maidstone, UK) was the most sensitive, consistent and practical variety of filter paper. Following optimization, the lower limit of detection for *Streptococcus pneumoniae* from dried mock CSF spots was 14 genomic equivalents (GE)/μL (interquartile range 5.5 GE/μL) or 230 (IQR 65) colony forming units/mL. A prospective clinical evaluation for *S. pneumoniae*, *S. suis* and *Neisseria meningitidis* was performed. Culture and PCR performed on fresh liquid CSF from patients admitted with a clinical diagnosis of meningitis (*n* = 73) were compared with results derived from dried CSF spots. Four of five fresh PCR-positive CSF samples also tested PCR positive from dried CSF spots, with one patient under the limit of detection. In a retrospective study of *S. pneumoniae* samples (*n* = 20), the median (IQR; range) CSF *S. pneumoniae* bacterial load was 1.1 × 10^4^ GE/μL (1.2 × 10^5^; 1 to 6.1 × 10^6^ DNA GE/μL). Utilizing the optimized methodology, we estimate an extrapolated sensitivity of 90%, based on the range of CSF genome counts found in Laos. Dried CSF filter paper spots could potentially help us to better understand the epidemiology of bacterial meningitis in resource-poor settings and guide empirical treatments and vaccination policies.

## Introduction

Bacterial meningitis is a life-threatening disease that carries significant mortality and considerable risk of severe neurological complications. In developing countries, such as the Lao People's Democratic Republic (Laos), the combined mortality rates of all causes of bacterial meningitis are likely to be >20% 1. Vaccine-preventable diseases account for the major portion of bacterial meningitis cases seen worldwide. As elsewhere in SE Asia, *Streptococcus pneumoniae*, *Streptococcus suis*, *Neisseria meningitidis* and *Haemophilus influenzae* account for the majority of cases of community-acquired bacterial meningitis in Laos (LOMWRU unpublished) 2–5. An improved understanding of the epidemiology of these diseases would support evidence-based national vaccination policies and empirical treatment guidelines. Vaccination programmes against *H. influenzae* type b, *S. pneumoniae* (seven and thirteen valent) and *N. meningitidis* (serotypes C, A, Y and W135) have had a profound impact on the incidence of bacterial meningitis cases in many regions 6–10.

In Laos, conventional bacteriological and molecular microbiological facilities for the diagnosis of central nervous system disease are confined to the capital city of Vientiane, and antibiotic treatment is often initiated prior to lumbar puncture 11. This may reduce the sensitivity of cerebrospinal fluid (CSF) culture by up to 33% 12. The polymerase chain reaction (PCR) has been shown to be superior to conventional methods, particularly when antibiotics have been administered 13.

Reliable procedures are necessary to preserve, transport and test CSF samples, frequently requiring a challenging and costly cold chain to be in place. Effective disease surveillance is thus severely hampered in resource-limited settings, such as those currently present in rural Laos, where procedures for appropriate laboratory diagnosis are suboptimal. Innovative, simple and inexpensive rapid diagnostic tests for detecting pathogen antigens in CSF hold promise 14–16. In West Africa, centralized PCR systems for CSF PCR have been developed 17, but it is likely that prolonged transport of CSF at high temperature will reduce sensitivity.

Blood dried onto filter paper to diagnose infectious diseases dates back to as early as 1939 18 and has proved to be an important tool for diagnosis, epidemiology and monitoring in settings with limited laboratory infrastructure. However, CSF has rarely been collected on filter paper. In the 1970s counter-immunofluorescence was used to detect capsular polysaccharide antigen in dried purulent CSF samples and more recently an enzyme-linked immunosorbent assay used to diagnose neurocysticercosis 19,20. Peltola *et al*. 21 successfully identified nucleic acids of *S. pneumoniae* and *H. influenzae* type b in CSF-impregnated filter paper strips after 8 months storage at room temperature in sealed plastic bags.

Until laboratory capacity is developed in rural Laos, filter paper could serve as a sample preservation method that can easily be delivered to a central laboratory for CSF PCR and therefore allow better understanding of the geographical epidemiology of bacterial meningitis. We investigated whether dried CSF spots would serve as a sensitive and reliable method for defining three of the major causative agents of bacterial meningitis in Laos: *S. pneumoniae*, *S. suis* and *N. meningitidis*. We investigated different types of filter paper and optimized the processing of dried CSF spots to obtain the maximum possible yield of nucleic acid. A prospective pilot study was conducted utilizing these optimized methods to compare PCR results on DNA extracted from dried CSF spots with routine liquid CSF and conventional bacterial culture.

## Methods

### Filter paper selection

Three varieties of filter paper were selected for performance evaluation: the Whatman Grade 903 (Cat. no. 10535097; Whatman), the Flinders Technology Associates (FTA) Micro Card™ (Cat. no. WB120210; Whatman) (referred to as FTA hereafter) and the FTA Elute Micro Card™ (referred to as FTA Elute hereafter) (Cat. No. WB120401; Whatman). Whatman 903 requires elution of the organism from the paper followed by lysis and DNA extraction. FTA and FTA Elute papers contain a proprietary mix of reagents that lyse cell walls but stabilize and bind nucleic acids. FTA paper requires a washing protocol, with the paper disc itself being used as a DNA template in the PCR.

A mock CSF was prepared by spiking tryptic soy broth (TSB) with a few discrete colonies of National Collection of Type Culture (NCTC) strains of either *S. pneumoniae* (NCTC 12977) or *N. meningitidis* (NCTC 10025) grown overnight on 5% goat blood agar. This was incubated for 4 h in 5–7% CO_2_ at 37°C and then diluted with sterile TSB to achieve a standardized initial concentration (optical density) using the Nanodrop 2000 (Thermo Scientific, Wilmington, DE, USA). Ten-fold serial dilutions were prepared and aliquots spotted onto filter paper (Whatman 903 and FTA, 125 μL/1 inch circle; FTA Elute, 40 μL/11 mm circle) and onto blood agar (100 μL) to determine the bacterial load (colony forming units/mL) for each dilution using a micropipette.

Optimization studies were performed using *S. pneumoniae* and to demonstrate inter-species reproducibility final methods were repeated with *N. meningitidis*.

### DNA extraction

Discs (3 or 8 mm) were punched out of the centre of a filter paper circle using skin biopsy punches (Stiefel, Maidenhead, UK) and transferred to 1.5-mL microcentrifuge tubes. Three and 8-mm punched discs would contain *c*. 3 μL and 20 μL of CSF, respectively. Biopsy punches were sprayed with 70% ethanol after each punch, dried with tissue paper and then punched into a stack of sterile filter paper a further five times to eliminate cross-contamination 22,23.

The FTA Elute manufacturer's instructions were followed with the following modifications: 1 mL of sterile water was used to wash the disc by pulse-vortexing three times for a total of 5 s. The DNA was eluted with sterile water after 22.5 min at 95°C, the mid-point of the manufacturer-recommended time period. FTA was processed following the manufacturer's instructions, except for the use of a slightly larger disc (3 mm in diameter rather than the recommended 2 mm). Whatman Grade 903 discs were processed following the QIAamp DNA Mini kit Dried Blood Spot Protocol (Qiagen, Crawley, UK) with the following modifications: elution was carried out in 180 μL of buffer ATL with proteinase K incubated at 56°C overnight.

Liquid CSF (*n* = 25) was initially processed using the QIAamp DNA Mini kit Blood or Body Fluids Protocol (Qiagen) after centrifuging 200 μL of CSF at 16 000 *g* and re-suspending the pellet in buffer ATL. Subsequently (*n* = 48), routine extraction was performed on 200 μL unspun CSF with the EZ1 Virus Mini Kit v2.0 for automated, simultaneous purification of viral DNA and RNA as well as bacterial DNA (Qiagen).

### Optimization of detection

To optimize DNA yield from filter paper, a series of experiments were performed in triplicate. Three different quantities of DNA eluate were used in the PCR: 3, 5 and 10 μL. A larger punch size of 8 mm (50.2 mm^2^) was compared with the standard size of 3 mm (7.1 mm^2^) (*c*. seven-fold increase in disc surface area). Finally, three different elution volumes were also investigated: 40, 80 and 145 μL. These experiments assumed an even distribution of pathogen DNA across the paper after application.

### Prospective evaluation: patients and samples

Patients admitted to Mahosot Hospital (*n* = 73), Vientiane, between July 2011 and March 2012 with a clinical diagnosis of meningitis, who underwent lumbar puncture, had fresh CSF spotted onto FTA Elute. Four circles were spotted with CSF and allowed to dry for 4 h before being stored in an air-conditioned laboratory at *c*. 20°C in sealed plastic bags containing silica granules to reduce humidity. PCR on dried CSF spots was subsequently compared with PCR performed on liquid CSF and routine culture.

### Culture

Patients' CSF samples were cultured on both 5% goat blood and chocolate agar (and MacConkey agar in those <1 year old) and incubated at 37°C in 5–7% CO_2_. Organism identification was performed following standard biochemical methods with additional confirmation provided using the API system (bioMerieux, Marcy l'Etoile, France).

### Molecular identification

Three separate real-time quantitative PCRs were performed for *S. pneumoniae*, *N. meningitidis* and *S. suis* (Table 1). Assays were performed as previously described, with slight modification 3,24,25. A 25 μL reaction volume contained 1x AmpliTaq Gold reaction buffer, 5 mM MgCl, 200 μM of each dNTPs (Applied Biosystems, Paisley, UK) and 1 U of AmpliTaq Gold polymerase (Applied Biosystems). Forward and reverse primers/probes were included with the following concentrations: *S. pneumoniae*, 200 nM/100 nM; *S. suis*, 400 nM/100 nM; *N. meningitidis*, 300 nM/25 nM. The cycling conditions were as follows: denaturation at 95°C for 10 min, followed by 40 cycles of amplification, each consisting of 95°C for 15 s and 60°C for 60 s. Three μL of DNA extracted from liquid CSF samples served as a template for each PCR assay. All runs were performed on a Rotor-Gene 6000 (Qiagen) and comprised at least two no-template controls as well as duplicates of serial dilutions of positive controls, which were quantitated using the Quant-iT PicoGreen assay (Invitrogen, Paisley, UK), corresponding to *c*. 10^3^, 10^2^, 10^1^ and 10^0^ genomic equivalents (GE)/μL.

**Table 1:** Oligonucleotide primers and probes for detection of *S. pneumoniae*, *S. suis* and *N. meningitidis*

	Sequence (5′–3′)	Reference
*S. pneumoniae*		Carvalho *et al*. 24
ctrA-Forward	ACGCAATCTAGCAGATGAAGCA	
ctrA-Reverse	TCGTGCGTTTTAATTCCAGCT	
ctrA Probe	ROX-GCCGAAAACGCTTGATACAGGGAG-BHQ2	
*S. suis*		Mai *et al*. 3
cps2J-Forward	GGTTACTTGCTACTTTTGATGGAAATT	
cps2J-Reverse	CGCACCTCTTTTATCTCTTCCAA	
cps2J Probe	FAM-TCAAGAATCTGAGCTGCAAAAGTGTCAAATTGA-TAMRA	
*N. meningitidis*		Corless *et al*. 25
ctrA-Forward	GCTGCGGTAGGTGGTTCAA	
ctrA-Reverse	TTGTCGCGGATTTGCAACTA	
ctrA Probe	FAM-CATTGCCACGTGTCAGCTGCACAT-BHQ1	

### Cerebrospinal fluid genome counts

Previously characterized frozen CSF samples collected from patients with bacterial meningitis admitted to Mahosot Hospital between 2005 and 2012 were identified retrospectively. These samples served to quantify the DNA loads in *S. pneumoniae* PCR-positive CSF samples, using external standards in a quantitative real-time PCR assay.

### Statistical analysis

Statistical analysis was performed with Stata (v11, College Station, TX, USA) and the figure created using GraphPad software (v6, La Jolla, CA, USA).

### Ethics

Lumbar punctures were performed as part of a routine diagnostic service if verbal (2003–2006) or written (2006–2011) consent was given by patients or their parents/guardian. Ethical clearance was granted by the Ethical Review Committee of the Faculty of Medical Sciences, National University of Laos, and the Oxford University Tropical Ethics Research Committee, Oxford, UK.

## Results

### Filter paper selection

**fig 1:**
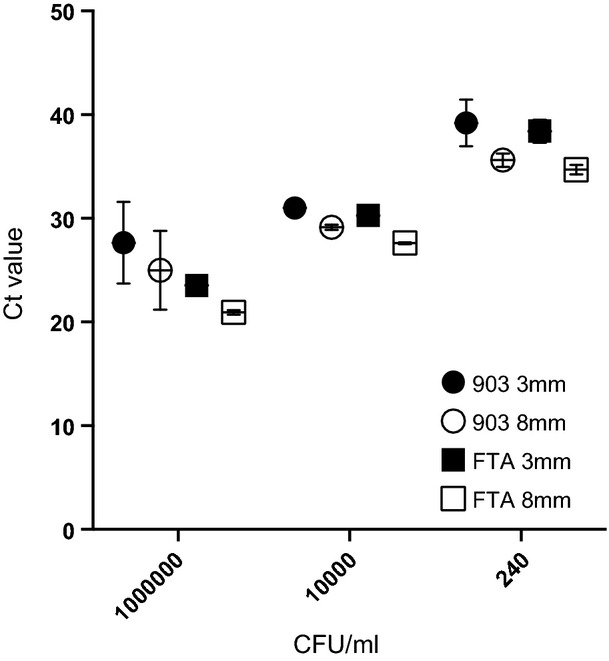
Median (and IQR) Ct values at different concentrations of *S. pneumoniae* (cfu/mL) in mock CSF on FTA Elute Micro Card and Whatman Grade 903 with two punch sizes.

Eight 10-fold dilutions of mock CSF were prepared ranging from 1 × 10^7^ cfu/mL to 1 cfu/mL. When applied to quantitative real-time PCR assay with external controls, the DNA templates eluted from FTA Elute consistently led to lower cycle thresholds than DNA eluted from Whatman 903 for the same mock CSF dilutions (median reduction 2.2 Ct values, IQR 1.68) (Fig. 1). Additionally, triplicate sets of Ct values for each mock CSF dilution were more consistent with FTA Elute (median Ct 0.42, IQR 0.37) compared with Whatman 903 (median Ct 4.44, IQR 3.53), suggestive of a more efficient and reliable DNA yield of *S. pneumoniae* from the FTA Elute (Fig. 1).

The FTA Micro Card was excluded after the first phase of experiments due to the limitation of paper size, which provided insufficient DNA template for performing the necessary number of single PCR assays required as a diagnostic routine for bacterial meningitis in a clinical setting.

### Optimization of detection

Increasing the quantity of DNA eluted from FTA Elute paper used in the PCR from 3 μL to 10 μL reduced the Ct values by a median of 0.94 (IQR 0.41), increasing the sensitivity of detection *c*. three-fold, without any negative effect on reaction efficiency.

In addition, increasing the punch size of FTA Elute from 3 mm (7.1 mm^2^) to 8 mm (50.2 mm^2^) reduced the Ct value by a median of 2.7 (IQR 0.55), equivalent to increasing the sensitivity of detection *c*. ten-fold (Fig. 1). Of the three elution volumes tested, the optimal volume was 40 μL, which led to the highest DNA concentration. An additional increase of the elution volume to 80 μL minimally decreased DNA concentration (median Ct value increase 0.22 (IQR 0.25)), whereas increasing the elution volume to 145 μL resulted in larger increases in Ct value (up to 1.1) and thus loss of sensitivity. As we aimed to detect several pathogens from dried CSF spots using single target real-time PCR assays, in order to have sufficient DNA template, we selected a final elution volume of 100 μL, accepting a very small reduction in sensitivity. The quantified limit of detection of the final conditions, using an 8 mm disc punched out of FTA Elute paper, with 100 μL elution volume and 10 μL in the PCR, was a median of 14 *S. pneumoniae* GE/μL spiked mock CSF (IQR 5.5), equivalent to a median mock CSF concentration of 230 cfu/mL (IQR 65). Studies to optimize the detection of *S. pneumoniae* from filter paper were reproducible using *N. meningitidis* (data not shown).

### Prospective evaluation of clinical samples

FTA Elute performed consistently with a high level of sensitivity throughout the optimization process and was therefore selected for further evaluation. A total of 73 dried CSF spots were prospectively collected from patients admitted with a clinical diagnosis of bacterial meningitis over a 9-month period. Dried CSF spots were stored at *c*. 20°C in sealed plastic bags containing silica granules for up to 8 months before being processed. Of these, 5/73 (6.8%) tested positive by quantitative real-time PCR on DNA extracted from liquid CSF (four *S. pneumoniae* and one *S. suis*) and 4/73 (5%) tested positive by quantitative real-time PCR on DNA from dried CSF spots (three *S. pneumoniae* and one *S. suis*). The positive samples were identical, resulting in a sensitivity of 80% (95% CI 29–97%) for PCR using FTA Elute. One sample was not detected by FTA Elute due to a low bacterial load of <10 GE/μL. Of the five positive samples, two were culture positive (both *S. pneumoniae*). No false-positives occurred, resulting in a specificity of 100% (95% CI 95–100%).

### Cerebrospinal fluid genome counts

Stored frozen (−80°C) DNA extracts of CSF samples from 20 well-characterized PCR-positive *S. pneumoniae* patients collected over a 7-year period were retrieved and their bacterial load quantitated. Genome equivalent counts in this population ranged widely from 1 × 10^0^ to 6.1 × 10^6^ DNA GE/μL (median 1.1 × 10^4^ GE/μL, IQR 1.2 × 10^5^ GE/μL) (Table 1). Given the bacterial loads from this small but representative sample of patients with *S. pneumoniae* meningitis presenting to a hospital in Vientiane, we would theoretically expect a positive result in 90% of the cases, applying the optimized methods. Very few positive *N. menigitidis* (*n* = 4)*, S. suis* (*n* = 5) and *H. influenzae* (*n* = 6) samples were available (data not shown).

## Discussion

In this study, we aimed to evaluate the use of dried CSF spots for the identification of three of the major causative agents of bacterial meningitis in Laos and to identify the role/potential limitations of using a filter paper-based surveillance approach to investigate the epidemiology of CNS disease aetiologies.

Our findings suggest that this can be accomplished using FTA Elute with the optimized methods, yielding an estimated sensitivity of 90% when compared with the ‘reference standard’ real-time diagnostic PCR assay on liquid CSF. Investigation with mock CSF suggested that FTA Elute gave the most consistent and sensitive results compared with Whatman 903. FTA paper discs used as a DNA template in the PCR proved impractical for testing several pathogens from a single dried CSF spot using single assays. We developed our methods using TSB that does not contain all the constituents of human CSF. However, the prospective evaluation of clinical samples confirmed our findings during the optimization process.

Despite using a larger than manufacturer-recommended punch size to increase the lower limit of detection, the volume of CSF used from paper is *c*. 10 times less than that used for routine PCR on liquid CSF (20 μL vs. 200 μL). Even this may be slightly overestimated due to possible diffusion outside the marked circle. As a result, despite the use of highly sensitive quantitative real-time PCR assays, those CSF samples with very low bacterial loads can fall under the limit of detection. The optimization studies demonstrated that, beyond the optimal cut-off, DNA concentrations in the eluate declined with increasing elution volumes. A small reduction in DNA concentration was accepted for the final methodology to allow sufficient PCR template to be available. Multiplex PCR can simplify the process of detecting multiple pathogens and potentially reduce costs. However, careful optimization must be performed to ensure that sensitivity and specificity are not reduced 26. We estimated that *c*. 230 cfu/mL or 14 GE/μL of CSF could be reliably detected by PCR from CSF dried on filter paper. In a study by La Scolea *et al*. 27 in the USA, 85% of CSF samples from children with bacterial meningitis had counts >10^3^ cfu/mL and 56% had counts >10^5^ cfu/mL. Samples collected in Laos over a 7-year period showed a wide range in GE/μL of *S. pneumoniae* with a median of 1.1 × 10^4^. This is consistent with other studies that report wide ranges and medians between 4.6 × 10^4^ and 5.7 × 10^5^ DNA copies/μL in Latin America, Australia and Malawi 21,28–30 (Table 2).

**Table 2:** Studies reporting median and ranges of *S. pneumoniae* copies/μL in CSF from different geographical regions

Country	Age	Study size	Median copies/μL	Range	Reference
Laos	All	20 patients	1.1 × 10^4^	12–6.1 × 10^6^	This manuscript
Venezuela & Paraguay	2 months–14 years	129 CSF samples	1.6 × 10^5^	5–9.3 × 10^6^	Peltola *et al*. 21
Latin America^a^	2 months–14 years	121 patients	4.6 × 10^4^	0–9.3 × 10^6^	Roine *et al*. 28
Malawi	2 months–16 years	82 patients	5.8 × 10^4^	44–6.2 × 10^5^	Carrol *et al*. 30
Australia	All	6 patients	1.1 × 10^4^	7.6–6 × 10^5^	van Haeften *et al*. 29

a Argentina, Dominican Republic, Brazil, Ecuador, Paraguay and Venezuela.

One single study reports the PCR identification of *H. influenzae* and *S. pneumoniae* from filter paper with sensitivities of 70% and 92% and specificities of 99% and 100%, respectively 21. CSF was taken from children in South America, transported to Finland at −20°C and subsequently thawed and spotted onto an unspecified Whatman filter paper. Sixty-four per cent of CSF samples were taken before antibiotics were administered. The range of DNA copies/μL for *S. pneumoniae* was 5 × 10^0^ to 9.2 × 10^6^, with a median of 1.6 × 10^4^, very similar to that seen in Laos (Table 1).

An additional limitation was that we were only able to prospectively test our methods on a limited number of samples and pathogens. Other studies have shown that *H. influenzae* is readily identified from dried CSF spots 21 and experiments using mock CSF detected *N. meningitidis* without difficulty. *Cryptococcus* spp. are also important causes of meningitis in Laos and preliminary investigations show that this would also be detectable by PCR from dried CSF spots (unpublished data). If other central nervous system pathogens were detectable in CSF using the optimized filter paper method, this would further increase the potential of using this methodology as a surveillance tool in areas without laboratory capacity.

We did not investigate the practicalities and suitability of collecting dried CSF spots in smaller regional hospitals and transporting these for analysis. Although the effects of humidity and storage conditions on dried CSF spots were not assessed, DNA was readily detected after 8 months storage in an air-conditioned room. A simple standard operating procedure was provided to laboratory staff for the preparation of dried CSF spots.

FTA Elute paper inactivates pathogens within a short period of application onto the paper 31, thus increasing the safety of handling and allowing easy transportation to a central laboratory in resource-limited settings. As a simple, inexpensive and reliable sample specimen preservation tool, the filter paper methodology has the potential to contribute to an improved understanding of the epidemiology of bacterial meningitis in resource-limited and rural regions of the world. This approach could support and guide vaccination programmes and empirical treatment protocols to reduce the morbidity and mortality associated with this life-threatening disease.

## Funding

Wellcome Trust of Great Britain.
